# Bacterial Resilience and Community Shifts Under 11 Draining-Flooding Cycles in Rice Soils

**DOI:** 10.1007/s00248-024-02468-y

**Published:** 2024-11-28

**Authors:** Anderson Santos de Freitas, Filipe Selau Carlos, Guilherme Lucio Martins, Gabriel Gustavo Tavares Nunes Monteiro, Luiz Fernando Wurdig Roesch

**Affiliations:** 1 Cell and Molecular Biology Laboratory, Center for Nuclear Energy in Agriculture, University of São Paulo, Piracicaba, SP Brazil; 2 Soil Department, “Eliseu Maciel” Agronomy Faculty, Federal University of Pelotas, Pelotas, RS Brazil; 3 Department of Microbiology and Cell Science, University of Florida, Gainesville, FL USA

**Keywords:** Rice cultivation, Soil resilience, Predicted functions, Wet-dry cycles

## Abstract

Flooded rice cultivation, accounting for 75% of global rice production, significantly influences soil redox potential, element speciation, pH, and nutrient availability, presenting challenges such as extensive water usage and altered soil properties. This study investigates bacterial community dynamics in rice soils subjected to repeated draining and flooding in Rio Grande do Sul, Brazil. We demonstrate that bacterial communities exhibit remarkable resilience (the capacity to recover after being altered by a disturbance) but cannot remain stable after long-term exposure to environmental changes. The beta diversity analysis revealed four distinct community states after 11 draining/flooding cycles, indicating resilience over successive environment changes. However, the consistent environmental disturbance reduced microbial resilience, causing the bacterial community structure to shift over time. Those differences were driven by substitutions of taxa and functions and not by the loss of diversity. Notable shifts included a decline in *Acidobacteria* and an increase in *Proteobacteria* and *Chloroflexi*. Increased *Verrucomicrobia* abundance corresponded with lower pH levels. Functional predictions suggested dynamic metabolic responses, with increased nitrification during drained cycles and a surge in fermenters after the sixth cycle. Despite cyclic disturbances, bacterial communities exhibit resilience, contributing to stable ecosystem functioning in flooded rice soils. These findings enhance our understanding of microbial adaptation, providing insights into sustainable rice cultivation and soil management practices.

**Supplementary Information:**

The online version contains supplementary material available at 10.1007/s00248-024-02468-y.

## Introduction

Outside of Asia, Brazil is the world’s top rice producer, with an annual production of 7866 thousand tons [1]. Most of the production comes from flooded rice cultivated during the summer. Dry land is used during the winter for pastures, wheat fields, and crop rotation with soybeans, pastures, and corn [2]. Shifts in soil conditions, such as changes in crop cover and field flooding, can significantly impact soil biogeochemistry and microbial dynamics, as microorganisms are susceptible to environmental disturbances [3].

Flooding alters the soil oxygen availability, altering soil biogeochemistry and fertility [4]. In the anoxic environment of floods, microorganisms can employ alternative final electron acceptors for organic matter degradation and energy acquisition, resulting in undesired byproducts like methane (CH_4_) and nitrous oxide (N_2_O), which are widely known greenhouse gases (GHG) [5]. Besides, rice production usually demands intensive nitrogen fertilization, which might result in increasing nitrous oxide emissions in the anaerobic conditions created by flood [6]. These undesired effects are mainly the result of changes in the soil microbial communities, emphasizing the need to understand better how microorganisms change during these cycles of draining and flooding.

Microorganisms are crucial in soil functions, including nutrient mineralization and solubilization, growth promotion, defense against phytopathogens, and controlling greenhouse gas flux [7, 8]. Changes in land use compel bacterial communities to reshape their composition and function in response to these factors [9]. Those changes may be reversible or lead to long-term alterations in the microbiome, depending on the source and duration of these environmental shifts [10, 11]. Studies in southern Brazil found that irrigated rice cultivation reduces soil microbial diversity compared to rotation with soybean, a rainfed crop [12], probably due to the low oxygen supply condition in this environment, which favors certain microbes with greater capacity to adapt to the environment. Flooding conditions have been shown to limit microbial activity in soils with a long history of flooding cycles [13]. Moreover, such limited environments create a habitat for a small community of microorganisms adapted to nutrient fluctuations, oxygen, and light availability [14]. While these adapted microorganisms contribute significantly to rice nutrition and production, there remains a considerable knowledge gap in understanding how successive flooding and drainage cycles shape the bacterial community in the soil.

Considering the annual cycles of flooding and draining of rice fields in Brazil, we hypothesize that repeated cycles of flooding and draining might unbalance the dynamic equilibrium of bacterial communities, leading to a loss of microbial diversity. Using next generation sequencing of the 16S rRNA gene and a controlled environment (without unpredictable field conditions), we investigate the impact of repeated soil inundation and drying cycles on bacterial community structure in a microcosm experiment.

## Materials and Methods

### Sample Collection and Chemical Analysis

Soil samples were collected in an agricultural area irrigated with rice cultivation for over 30 years in Brazil’s Rio Grande do Sul state. The area was located in Camaquã city (30°51′5″ S; 51°48′46″ W), and the soil was classified as Entisol according to the Soil Survey Staff [15].

We collected 20 kg of arable soil layer (0–20 cm). Soils were air-dried for 10 days in 7-cm-high trays, then sieved through a 4-mm sieve, and a sub-sample was taken for chemical analysis (Table 1). Organic matter was measured by the Walkley–Black [16]. Electrical conductivity and pH were measured in water (soil: water 1:1 and 1:5, respectively). Al^3+^, ammonium, nitrate, calcium, and magnesium were extracted by KCl 1 mol L^−1^. Phosphorus and potassium were extracted by Mehlich-1. The analyses were carried out following the recommendations of Tedesco and colleagues [17].

**Table 1:** Initial chemical composition of soils

Variable	Value
Organic matter (%)	6.82 ± 0.28
Clay (g.kg^−1^)	34 ± 1.0
Silt (g.kg^−1^)	35 ± 1.2
Sand (g.kg^−1^)	31 ± 0.9
pH (H_2_O)	4.42 ± 0.03
Electrical conductivity (µS cm^−1^)	346.5 ± 14.7
Al^3+^ (cmol_c_ dm^−3^)	3.87 ± 0.08
Ammonium (mg kg^−1^)	55.73 ± 1.37
Nitrates (mg kg^−1^)	28.82 ± 1.46
P (mg dm^−3^)	40.73 ± 1.34
K (mg dm^−3^)	144.6 ± 5.1
Ca (mg dm^−3^)	8.69 ± 0.15
Mg (mg dm^−3^)	3.55 ± 0.26

Values are shown as mean ± standard deviation

### Microcosm Experiment Design

In a completely randomized design, pots were filled with 3 L of homogenized soil with four replicates. The soil-filled pots were incubated in a biochemical oxygen demand (BOD) incubator at 20 °C without light during the experiment. Each cycle of draining and flooding lasted 30 days. The first cycle was conducted with the soil immediately after adjusting the humidity to the field capacity (cycle 01—drained). During the drained cycles, the pots were weighted every 2 days, and distilled water was added when necessary to keep the soils at the field capacity. After 30 days, the pots were irrigated with distilled water till all the soil pores were filled and a 2-cm water sheet formed over the soil surface (cycle 02—flooded). The water level was maintained at 2 cm during the flooding cycle. After another 30 days, the pots were drained to remove the free water. A drainage system was created for each pot using a perforated PVC pipe wrapped in a mesh of 200 microns. A hose was connected to this pipe and attached to a small pump to extract the water. The remaining moisture was gradually removed until field capacity (cycle 03—drained). Those procedures were repeated until we completed 11 cycles of draining and flooding, ending in cycle 11—drained.

### Sampling, DNA Extraction, and Library Preparation

After each cycle, we collected 250 mg of soil using a sterile micro spoon for DNA extraction and measured pH (DM 23-Digimed) and electrical conductivity (Digimed Dm-3) at the end of the cycle before proceeding with the subsequent flooding or drainage. All samples were kept at − 20 °C until the DNA extraction. We extracted the DNA from all pots at the end of all cycles (4 pots/replicates in 11 cycles), totaling 44 DNA samples.

DNA extraction was performed with the PowerSoil DNA Isolation Kit™ (Qiagen, Hilden, Germany) following the manufacturer’s instructions. DNA quality was measured using a NanoVue™ spectrophotometer (GE Healthcare, Chicago, IL, USA). All DNA samples were then stored at − 80 °C for downstream analysis.

For microbial identification, the V4 region from the 16S rRNA gene was amplified by PCR using the bacterial/archaeal primers 515F and 806R [18]. Multiple samples were amplified using barcoded primers linked to the Ion adapter “A” sequence (5′-CCATCTCATCCCTGCGTGTCTCCGACTCAG-3′) and Ion adapter “P1” sequence (5′-CCTCTCTATGGGCAGTCGGTGAT-3′) to obtain a sequence of primer composed for A-barcode-806R and P1-515F adapter and primers. Each 25 µL reaction consisted of 2 µL of Platinum® Taq DNA High Fidelity Polymerase (Invitrogen, Carlsbad, CA, USA), 4 µL 10X High Fidelity PCR Buffer, 2 mM MgSO4, 0.2 mM dNTP’s, 0.1 µM of both the 806R barcoded primer and the 515F primer, 25 µg of Ultrapure BSA (Invitrogen, Carlsbad, CA, USA), and approximately 50 ng of DNA template.

The resulting PCR products were purified with the Agencourt® AMPure® XP Reagent (Beckman Coulter, Brea, CA, USA), and the PCR product’s final concentration was quantified using the Qubit Fluorometer kit (Invitrogen, Carlsbad, CA, USA) following manufacturer’s recommendations. The reactions were then combined into equimolar concentrations to create a mixture of each sample’s 16S gene amplified fragments. This mixture was used for library preparation with the Ion OneTouch™ 2 System with the Ion PGM™ Template OT2 400 Kit Template (Thermo Fisher Scientific, Waltham, MA, USA).

Sequencing was performed using Ion PGM™ Sequencing 400 on Ion PGM™ System using Ion 318™ Chip v2 with a maximum of 40 samples per microchip. All relevant data are fully available without restriction. Raw sequences were deposited in the NCBI Sequence Read Archive under the BioProject ID PRJNA1079370.

### Sequence Processing and Data Analysis

All processing and analyses were performed in the R environment [19]. Raw reads were analyzed following the recommendations of the Brazilian Microbiome Project [20] and the DADA2 pipeline [21]. Primers, adapters, and barcodes were removed from the raw sequences. The multiplexed sequences were filtered and trimmed using the default settings for Ion Torrent sequences (maxEE = 2; trimLeft = 15). Then, paired reads were merged, and chimeras were removed. The high-quality sequences (Q ≥ 30) of 300 bp were taxonomically assigned against the SILVA rRNA database version 138.1 [22]. The sampling effort was measured by Good’s coverage [23].

We used the Shapiro–Wilk test to compare electrical conductivity (EC) and pH during the cycles. If the null hypothesis was rejected (indicating that the data were not normally distributed), we tested these variables for differences using the Kruskal–Wallis and post hoc Dunn tests. To analyze the ASVs’ abundance, we considered the compositional nature of the data [24] and center log-ratio transformed the sequence counts for all comparative analyses, except for observed diversity when we only used the rarefied original data [25, 26].

Alpha diversity indices (Observed number of ASVs and InvSimpson) were calculated using the *vegan* package [27]. Beta diversity analyses were performed using PERMANOVA in the *vegan* package and plotted using Principal Coordinates ordination and Euclidean distance. The relative abundance was calculated and plotted using the *microeco* package [28]. The overall distribution of phyla per cycle was performed using the *microbiome* package [29]. The ALDEx2 package was used to calculate differential abundance in genera composition at each cycle, considering only *p*-values calculated by Wilcoxon’s test equal to or lower than 0.01 and effect size equal to or higher than 2.00 as significant [30]. Finally, we predicted the putative functional profile of bacterial communities using the FAPROTAX tool [31] in the *microeco* package. The R code used in this work is available at https://github.com/FreitasAndy/Draining_Flooding.

## Results

In this study, we obtained a total of 1,434,127 high-quality sequences. The average number of sequences per sample was 33,352 (median = 29,712). This sequencing depth was sufficient to achieve a representative sampling of the soil bacterial community, as indicated by Good’s coverage (> 99%). One of the 44 samples sequenced (from cycle 02) had insufficient reads and was excluded from the dataset. Consequently, 43 samples were included in the subsequent analysis.

### Microbial Diversity Does Not Correlate with pH and Electrical Conductivity

**Fig. 1:**
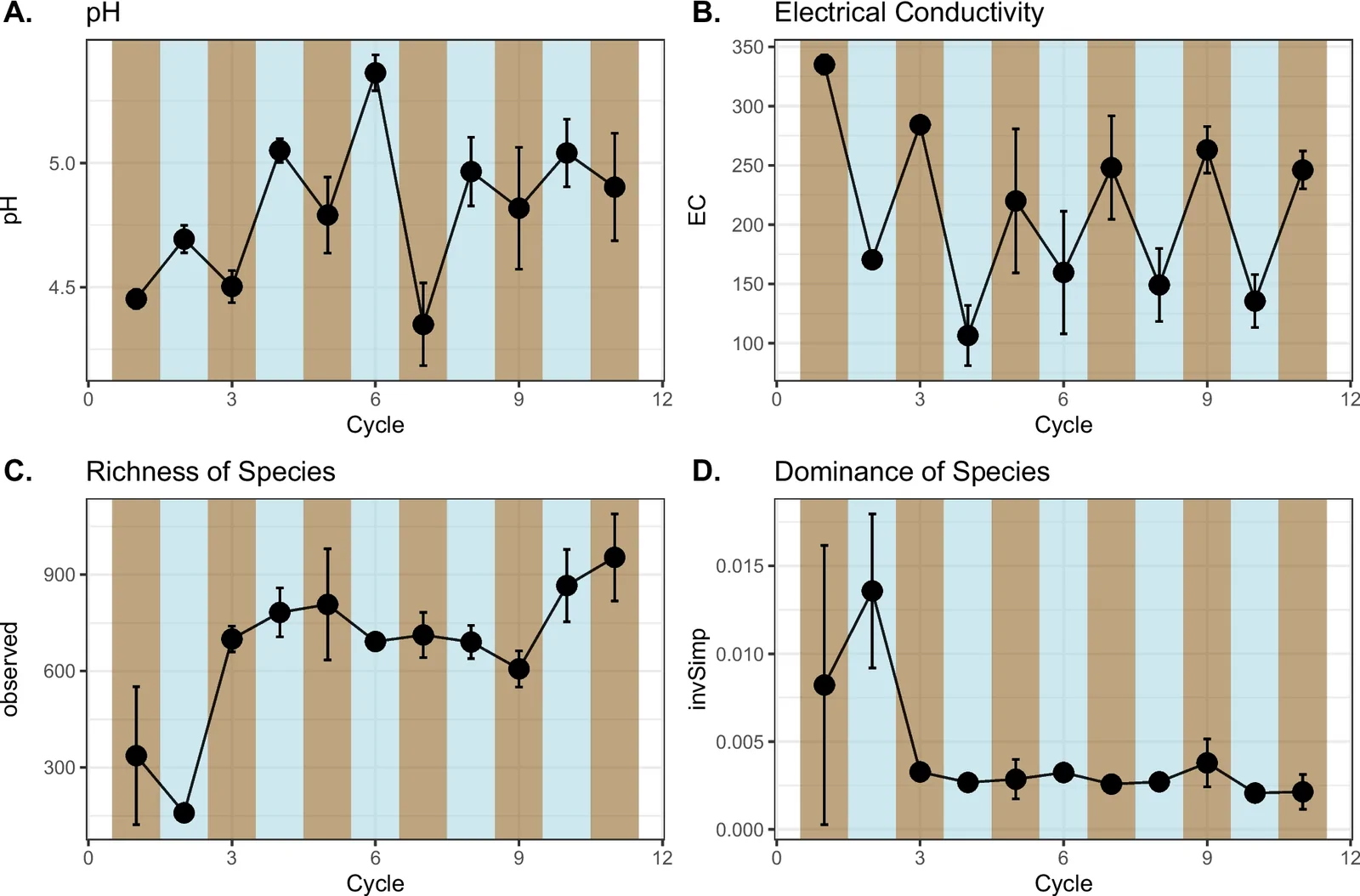
Dynamics of soil attributes and alpha diversity in cycles of draining and flooding in rice cultivation samples. Brown panels represent drained cycles, whereas blue panels represent flooded cycles. **A** Soil pH variation over time. **B** Soil electrical conductivity over time. **C** Soils observed diversity. **D** Soils inverse the Simpson index over time. Each dot represents the mean of that variable in that cycle. Whiskers represent the standard deviation of the mean of that variable in that cycle

The soil electrical conductivity (EC) and the pH varied over the flooding and drying cycles. Flooding increased the pH and reduced the EC in all cycles. (Fig. 1A and B). The pattern from the first to the last cycle remained similar, with these variables returning to levels close to the initial values even after 11 cycles of draining and flooding.

The measures of bacterial alpha diversity did not correlate with the pH and EC patterns. The bacterial richness increased during the experiment, achieving the highest value in the last cycle. The increase occurred mainly between cycle 02 (flooded) and cycle 03 (drained) (Fig. 1C). The mean values of bacterial dominance were higher during the first two cycles but decreased to levels lower than the initial ones during all subsequent flood and soil drainage cycles (see Fig. 1D).

### Flooding and Drying Cycles Cause Beta Diversity to Change Over Time

**Fig. 2:**
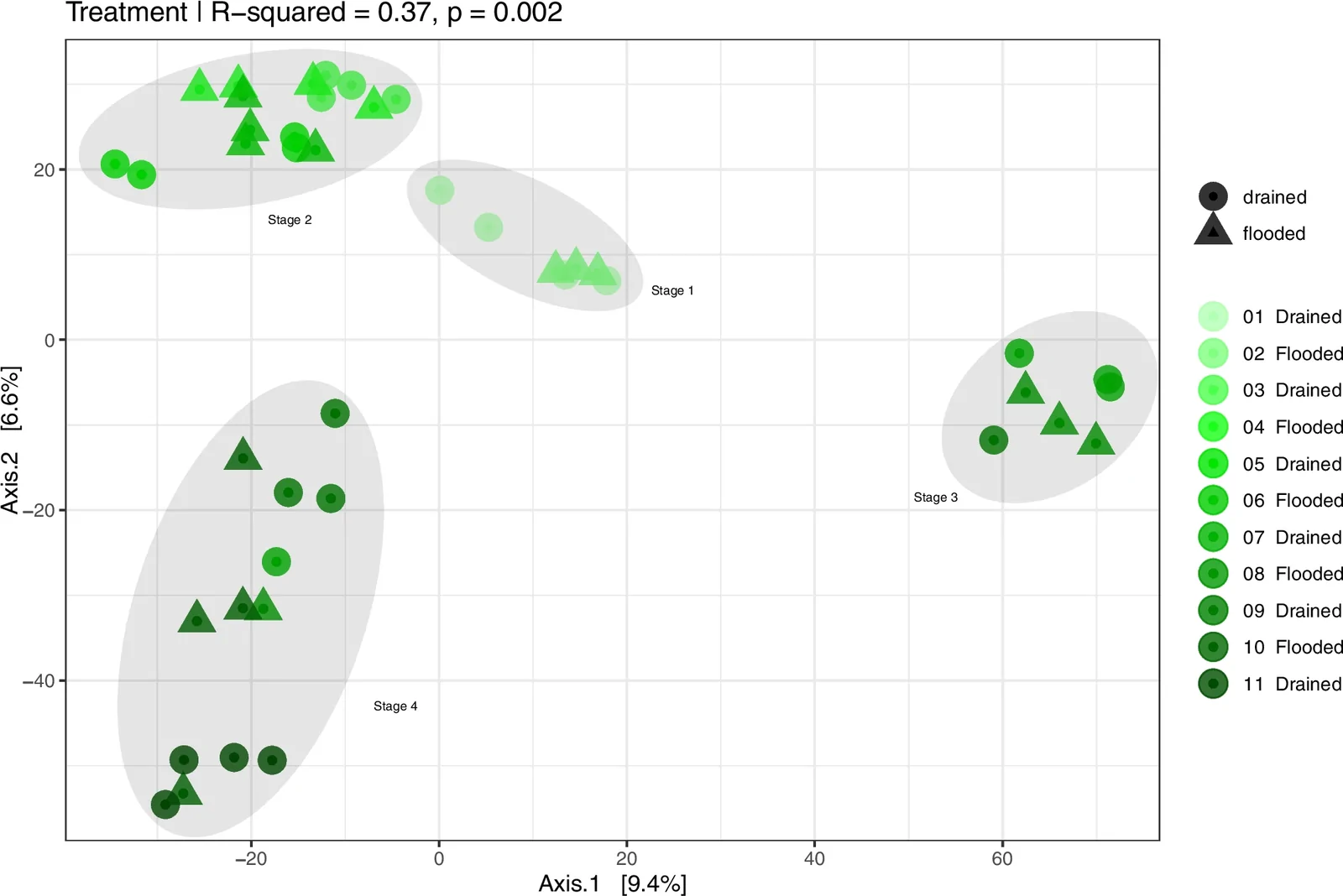
Overall comparisons of bacterial communities based on principal coordinates analysis (PCoA) calculated by the Euclidean distance of the Centered Log Ratio transformed data, depicting clusters of bacterial communities in 43 samples from soils used for rice cultivation in 11 cycles of draining and flooding. Each point represents a microbial community. Points closer to each other represent similar bacterial communities, while points farther from each other represent dissimilar ones. The statistical significance of sample groupings was tested by PERMANOVA using distance matrices as the primary input (for more details on PERMANOVA, please see Supplementary Table 1)

The bacterial community structure changed throughout the flooding and drying cycles, revealing four distinct groups called community stages over the experimental period (see Fig. 2). The first and second cycle samples were grouped, composing the initial community stage. The second stage included soil samples from the third to the sixth cycle. The communities clustered in a distinct and distant group in the seventh and eighth cycles. Finally, the last three cycles were grouped in another community cluster, indicating consistent changes in the community structure over time. The pairwise PERMANOVA showed changes in beta diversity across all cycles. The changes were more significant in the initial and less pronounced in the final cycles. This suggests that bacterial communities adapt to the changes caused by the draining and flooding cycles, becoming less affected over successive cycles. (See Supplementary Table 1 for details). On average, the *R*^2^, which represents the percent of the variation in the bacterial community explained by the cycles of draining and flooding, was 36% (*p*-value = 0.001) in the soils (Supplementary Table 1). The impact of these cycles on microbial beta diversity was significant in 74% of the pairwise comparisons.

### Microbial Differential Abundance

**Fig. 3:**
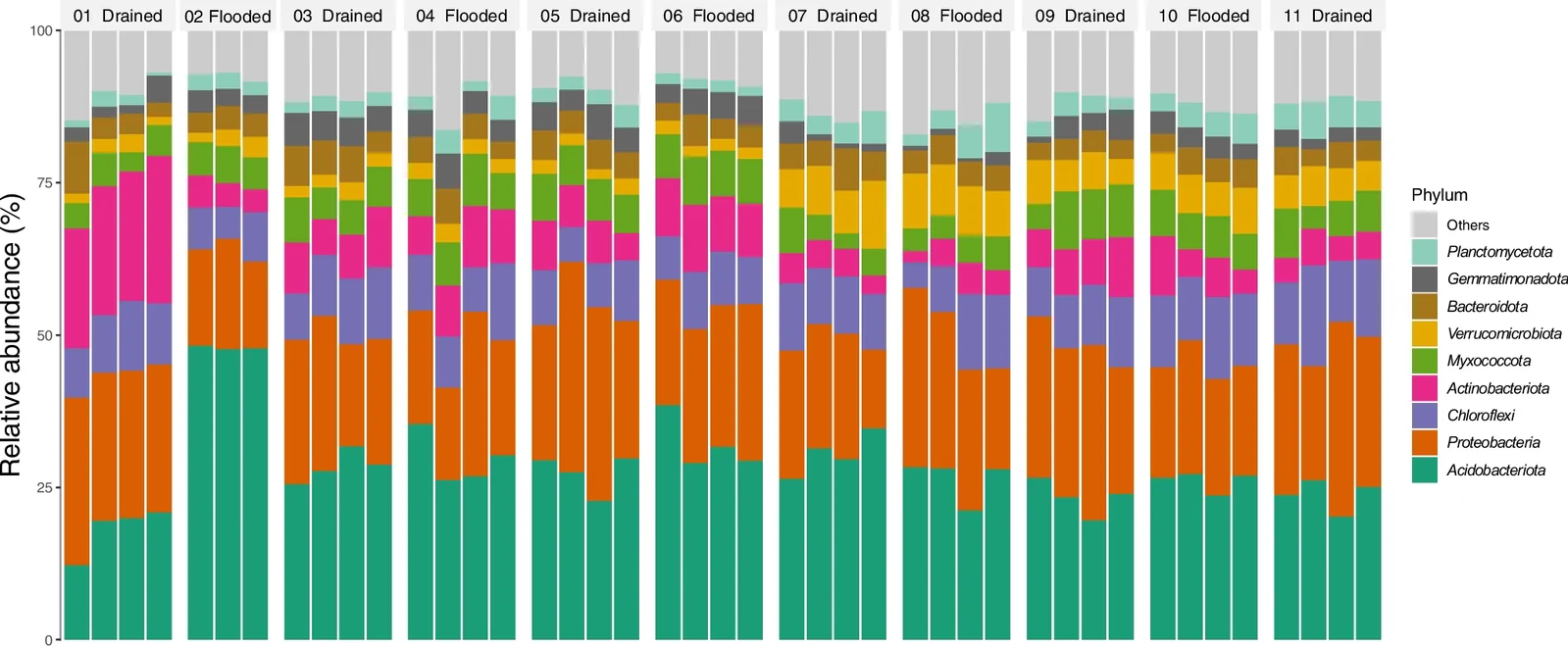
Phyla abundance of the top 10 taxa in rice soil during draining and flooding cycles. Cycle 01 was drained, cycle 02 was flooded, and then successively until cycle 11, which was drained

The differences in bacterial communities between the second and third cycles, as shown in alpha and beta diversity (Figs. 1C, D and 2), were mainly caused by a decrease in *Acidobacteria* and an increase in *Proteobacteria* and *Chloroflexi* (Fig. 3). These changes were linked to fluctuations in soil pH and EC, which affected nutrient availability and the composition of the bacterial community.

After the first two cycles, the abundance of phyla remained consistent until the 7th cycle, at which point we observed a continuous increase in *Verrucomicrobia* until the end of the experiment. This observation is consistent with the beta diversity analysis (see Fig. 2). In the final stage, beginning in cycle 09, there was a slight recovery in the abundance of *Acidobacteria* and *Myxococota*. The first community stage was also marked by a decrease in *Terracidophilus* from the first to the second cycle (Table 2). However, the most significant differences were observed after the third cycle, following the initial draining event in the experiment. Thirty-three genera have increased, including *Haliangium*, *Conexibacter*, and *Gemmatiomonas*, each with an effect size greater than 5. *Terracidophilus* has also increased again after this transition (Table 2 and Supplementary Figure S1).

**Table 2:** Differential abundance of soil microbial genus among cycles of draining and flooding

Cycle	Phylum	Genus	Status	Effect	*p*-value
Drained to flooded (1–2)	*Acidobacteriota*	*Terracidiphilus*	Increased	2	0.008
Flooded to drained (2–3)	*Acidobacteriota*	*Bryobacter*	Increased	2.08	0.001
		*Terracidiphilus*	Increased	2.03	0.003
	*Actinobacteria*	*Conexibacter*	Increased	5.38	< 0.001
		*Nocardioides*	Increased	3.47	< 0.001
		*Mycobacterium*	Increased	2.4	< 0.001
		*Nocardia*	Increased	2.15	< 0.001
	*Armatimonadota*	*Chthonomonas*	Increased	2.68	< 0.001
	*Bacteroidota*	*Flavisolibacter*	Increased	2.69	0.001
	*Bdellovibrionota*	*Bdellovibrio*	Increased	3.46	< 0.001
	*Chloroflexi*	*HSB OF53-F07*	Increased	3.54	< 0.001
		*FCPS473*	Increased	2.73	< 0.001
	*Cyanobacteriota*	*Vampirovibrio*	Increased	2.65	< 0.001
	*Elusimicrobiota*	*Endomicrobium*	Increased	2.25	< 0.001
	*Gemmatimonadota*	*Gemmatimonas*	Increased	5.22	< 0.001
	*Myxococcota*	*Anaeromyxobacter*	Increased	2.7	< 0.001
	*Nitrospirota*	*Nitrospira*	Increased	2.08	< 0.001
	*Proteobacteria*	*Haliangium*	Increased	5.73	< 0.001
		*Rhodoplanes*	Increased	4.53	< 0.001
		*Acidibacter*	Increased	3.99	< 0.001
		*MND1*	Increased	3.72	< 0.001
		*Noviherbaspirillum*	Increased	3.37	< 0.001
		*Ellin6067*	Increased	3.27	< 0.001
		*Hyphomicrobium*	Increased	2.93	< 0.001
		*Rhodomicrobium*	Increased	2.83	< 0.001
		*Polycyclovorans*	Increased	2.79	< 0.001
		*Sterolibacterium*	Increased	2.78	< 0.001
		*mle1-7*	Increased	2.57	< 0.001
		*Candidatus Ovatusbacter*	Increased	2.51	< 0.001
		*Sphingomonas*	Increased	2.43	0.001
		*Sulfurifustis*	Increased	2.28	< 0.001
	*Thermoproteota*	*Candidatus Nitrocosmicus*	Increased	2.99	< 0.001
	*Verrucomicrobiota*	*Candidatus Udaeobacter*	Increased	2.11	< 0.001
Drained to flooded (3–4)		None	-	-	-
Flooded to drained (4–5)	*Proteobacteria*	*Panacagrimonas*	Increased	3.48	< 0.001
Drained to flooded (5–6)	*Actinobacteria*	*Actinoplanes*	Increased	3.43	< 0.001
Flooded to drained (6–7)	*Omnitrophica*	*Candidatus Omnitrophus*	Increased	2.37	< 0.001
Drained to flooded (7–8)		None	-	-	-
Flooded to drained (8–9)		None	-	-	-
Drained to flooded (9–10)		None	-	-	-
Flooded to drained (10–11)		None	-	-	-

No genera were considered significantly decreased between cycles according to the parameters used

After these three cycles, however, the differential abundance over cycles was minimal. Only three events happened: an increase in *Panacagrimonas* after draining in cycle 05, an increase in *Actinoplanes* after flooding in cycle 06, and an increase in *Candidatus Omnitrophus* after draining in cycle 07.

### Abundance of Putative Functions

**Fig. 4:**
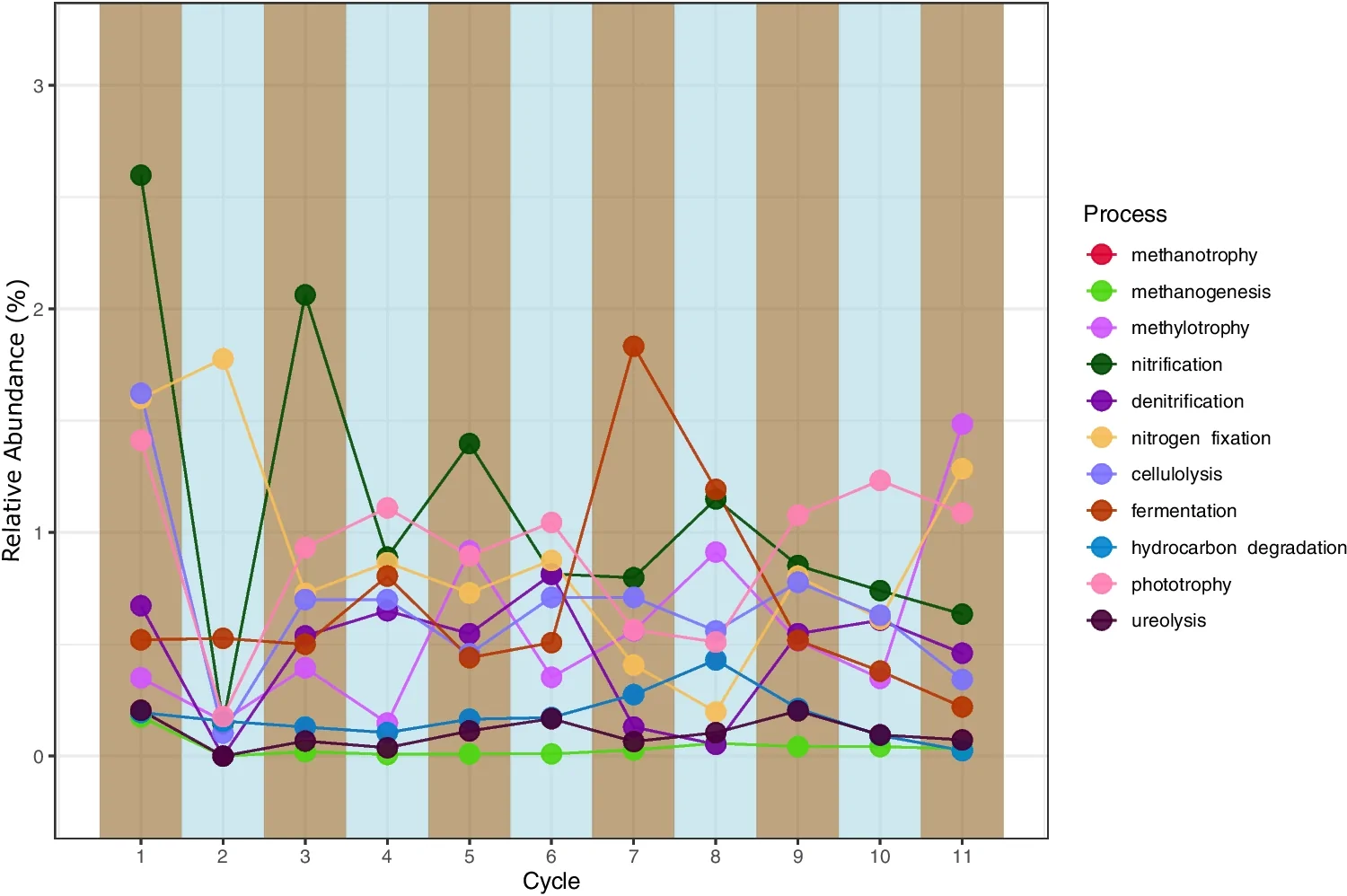
The abundance of putative predicted functions in the bacterial community of soils. Each panel represents one cycle of draining or flooding, with brown panels representing drained and blue panels representing flooded cycles

The changes in expected functions during the cycles showed variations between flooded and dry cycles (see Fig. 4). However, functions related to methanogenesis and ureolysis showed fewer variations between those two conditions. After the entire experiment, there was a trend of decreasing potential nitrification in flooded cycles, resulting in a lower overall level. Interestingly, there was a significant increase in putative fermenter microorganisms in the seventh cycle (drained), which was immediately lost in the following cycles. Additionally, we observed increased phototrophy in the three final cycles (Fig. 4).

## Discussion

This study investigated the shifts in the bacterial community over draining and flooding cycles of soils from rice fields. Soil water content plays an essential role in shaping the composition of soil bacterial communities in flooded rice systems. We hypothesized that repeated flooding and draining cycles might unbalance bacterial communities’ dynamic equilibrium, causing a loss of microbial diversity. The results rejected that hypothesis, indicating the diversity was not affected by successive draining and flooding cycles. However, while the bacterial community resisted the stress imposed by the flooding and drying cycles, remaining unaltered for a few cycles, the consistent environmental disturbance reduced the microbial resilience, causing shifts in the bacterial community structure over a long time. Those differences were driven by substitutions of taxa and functions rather than by the loss of diversity.

Bacterial communities may adapt to changes in redox conditions caused by flooding/drainage cycles, conferring greater physiological resilience against these disturbances [32]. In combination with larger population size, the community becomes less susceptible to diversity losses from disturbances, allowing faster recovery and recolonization of vacant niches after disturbances, preventing long-term diversity declines. However, this effect may depend on several factors. For instance, forest soils tend to exhibit higher resilience in bacterial structure under drought-wet stresses, while agricultural soils are more sensitive [33]. Many prokaryotic groups can enter a dormant state during drought but reactivate after rewetting [34]. Typically, microbial responses to draining-flooded cycles vary from a resilience, where recovery is fast, to a sensitive response, where recovery occurs only after a lag period with no initial growth [35].

Bacterial alpha diversity did not correlate with pH and EC variations, emphasizing the complexity of factors influencing microbial richness and dominance. The initial decrease in bacterial richness followed by an increase in the third cycle implies a dynamic response to the environmental stress imposed by flooding and drying cycles. Additionally, the soil was collected in the field and transported to a controlled environment, which might have caused disturbances that could lead to this higher imbalance in the first cycles [36]. Nonetheless, the decrease in bacterial dominance in the third cycle further suggests a potential recovery or adaptation phase (Fig. 1). Although soil disturbances usually suppress soil biological activity, the indigenous microbes have mechanisms to adapt to the new conditions, especially if these fluctuations in soil conditions (i.e., pH, EC, and water content) follow a pattern, as observed in this experiment [37, 38].

Although the environmental conditions did not affect the alpha diversity, the beta diversity analysis revealed significant bacterial community structure shifts over time. The four community states observed during the experiment imply a degree of microbial resilience, as no immediate microbial structure changes were observed following each flooding/drying cycle. Significant changes were only observed after at least 2–3 flooding/drying cycles. Such events are known to shape bacterial communities by selecting taxa with high functional plasticity [39]. The temporal dynamics and the eventual convergence of bacterial communities into a stable new state suggest resilience and a capacity for adaptation to the imposed stressors, as seen in the shifts in microbial composition at the phylum level (Fig. 2). *Acidobacteria*, *Proteobacteria*, and *Chloroflexi* were specific microbial groups mostly affected by long-term environmental changes. *Acidobacteria* is well adapted to acidic and anaerobic environments, which are precisely the conditions in the soil after flooding and can be associated with an increase in the genus *Terracidophilus* (Table 2) [40]. Disturbed bacterial communities change in diversity and structure [8]. Still, they tend to establish some patterns if the new disturbances have equal or lower intensity and if the carbon levels remain similar [41]. Under drying conditions, the increase in oxygen availability allows many aerobic microbes to grow. These taxa were the most adapted to the new environmental conditions and could keep occupying the niches after new cycles of flooding and draining [42].

The phylum *Verrucomicrobia* increased after cycle 07, causing the changes observed in the community stage, as shown in Fig. 2. The plasticity of this phylum could explain this process, which is adapted to the climatic fluctuations in the Pampa biome and related to high carbon content [9, 43]. Cycle 07 also marked the transition from stage 2 to stage 3 in beta diversity (Fig. 2), and the significant change in differential abundance was driven by the *Candidatus Omnitrophus* from the *Verrucomicrobia* phylum. This genus is related to metabolic interactions with hydrogen-consuming partners and obligate fermentative heterotrophy [44], which matches the increase in putative fermentation (Fig. 4) and the lowest pH found in a flooded cycle during the experiment, both at cycle 07 (Fig. 2).

Finally, the last three cycles were marked by a decrease in putative fermenters and nitrifiers (Fig. 4) and pH stabilization (Fig. 1A). The conditions became more suitable and less restrictive, increasing alpha diversity (Fig. 1C). The increase in *Myxococota* members can also be related to this process since this phylum is mainly composed of aerobic spore-forming species in soil that will be unable to grow in a more competitive environment with a lack of oxygen [45].

In short, our data rely on the concept of microbial functional redundancy. In complex microbial communities such as those found in rice soils, multiple taxa often share overlapping functional roles. This redundancy allows for the maintenance of ecosystem functionality even as individual taxa are replaced, or their relative abundances fluctuate [46]. The core community maintains essential soil processes, such as nitrogen cycling and organic matter degradation, highlighting the importance of microbial functional potential as a critical component of ecosystem resilience beyond simple measures of taxonomic diversity.

Also, some microbial taxa may enter a state of dormancy during alternating wet and dry conditions characterized by reduced metabolic activity. These dormant microbes can be reactivated when environmental conditions become favorable, such as during soil reoxygenation following drainage [47]. This dormancy-response mechanism ensures the survival of sensitive taxa, which can later contribute to community recovery. Moreover, the selection pressure imposed by fluctuating conditions may favor microbial taxa with specialized metabolic strategies, such as facultative anaerobes and microbes capable of switching between metabolic pathways (e.g., aerobic and anaerobic respiration) [39]. Understanding these microbial survival strategies is vital for predicting long-term soil ecosystem responses to cyclical environmental stressors like those in flooded rice systems.

## Conclusions

Despite the limitations that arise from microcosms, as they do not reflect the entire dynamic of natural environments, our findings suggest a trend toward a microbial community adapted to compete in either flood or dry soils after repeated cycles of draining and flooding. After the first cycle, a noticeable increase in bacterial dominance was observed, gradually decreasing with continuous wet-dry cycles. We could observe a higher distinction between the distinct soils and their microbial community in the initial cycles, which became increasingly similar with each wet-dry cycle. Long-term exposure to wet-dry cycles increased bacterial richness, suggesting a competitive environment for the soil bacterial community. Additionally, predicted functions related to oxygen-dependent activities exhibited a decreasing trend, indicative of a microbial community more adapted to continuous cycles of draining and flooding. However, in situ studies are needed to confirm the observed trends in this study further.

## Supplementary Information

Below is the link to the electronic supplementary material.

Supplementary file1 Heatmap showing the abundance of the top 40 genera in rice soils submitted to cycles of draining and flooding. (PNG 80 KB)

Supplementary file2 (DOCX 18 KB)

## Data Availability

Sequence data that support the findings of this study have been deposited in the NCBI Sequence Read Archive under the BioProject ID PRJNA1079370.
