# Supplementary figures and images for: Bacterial Resilience and Community Shifts Under 11 Draining-Flooding Cycles in Rice Soils
Source: Microb Ecol. 2024 Nov 28;87(1):149. doi: 10.1007/s00248-024-02468-y (PMC11602802; doi:10.1007/s00248-024-02468-y)

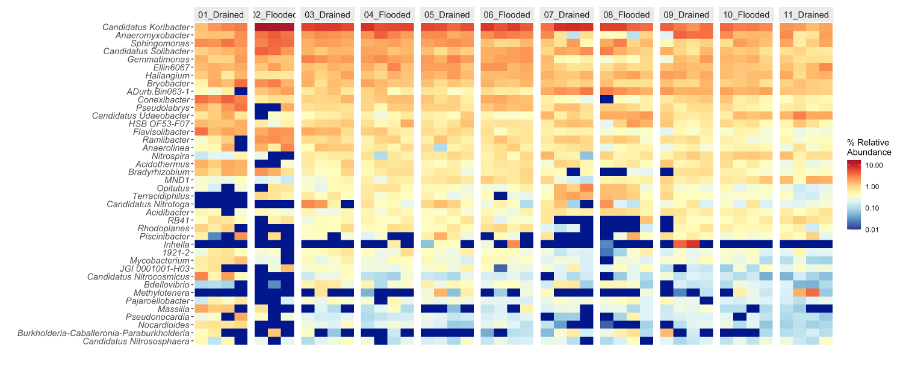

Supplement: Supplementary file 1 — Supplementary file1 Heatmap showing the abundance of the top 40 genera in rice soils submitted to cycles of draining and flooding. (PNG 80 KB) [file 248_2024_2468_MOESM1_ESM.png]
