# Supplementary material for: Bacterial Resilience and Community Shifts Under 11 Draining-Flooding Cycles in Rice Soils
Source: Microb Ecol. 2024 Nov 28;87(1):149. doi: 10.1007/s00248-024-02468-y (PMC11602802; doi:10.1007/s00248-024-02468-y)
Supplement: Supplementary file 2 — Supplementary file2 (DOCX 18 KB) [file 248_2024_2468_MOESM2_ESM.docx]

**Supplementary Table 1.** The R^2^ values from the pairwise PERMANOVA. Numbers followed by * indicate a p-value <= 0.05.

|  | | Pairwise PERMANOVA | | | | | | | | |
| --- | --- | --- | --- | --- | --- | --- | --- | --- | --- | --- |
|  | Flooded 2 | Drained 3 | Flooded 4 | Drained 5 | Flooded 6 | Drained 7 | Flooded 8 | Drained 9 | Flooded 10 | Drained 11 |
| Drained 1 | 0.20* | 0.29* | 0.24* | 0.21* | 0.23* | 0.23* | 0.24* | 0.21* | 0.22* | 0.31* |
| Flooded 2 |  | 0.34* | 0.26* | 0.25* | 0.27* | 0.27* | 0.28* | 0.26* | 0.25* | 0.35* |
| Drained 3 |  |  | 0.20* | 0.19* | 0.21* | 0.24* | 0.27* | 0.27* | 0.27* | 0.35* |
| Flooded 4 |  |  |  | 0.16* | 0.17* | 0.21* | 0.23* | 0.23* | 0.24* | 0.32* |
| Drained 5 |  |  |  |  | 0.14 | 0.18* | 0.21* | 0.21* | 0.22* | 0.31* |
| Flooded 6 |  |  |  |  |  | 0.19* | 0.21* | 0.21* | 0.23* | 0.31* |
| Drained 7 |  |  |  |  |  |  | 0.16* | 0.16* | 0.20* | 0.29* |
| Flooded 8 |  |  |  |  |  |  |  | 0.14 | 0.21* | 0.29* |
| Drained 9 |  |  |  |  |  |  |  |  | 0.20 | 0.29* |
| Flooded 10 |  |  |  |  |  |  |  |  |  | 0.16* |
| Drained 11 |  |  |  |  |  |  |  |  |  |  |
|  | PERMANOVA under reduced model – draining and flooding cycles | | | | | | | | | |
|  | Df | Sum of Sqs | R2 | F | Pr(>F) |  |  |  |  |  |
| Cycles | 10 | 147820 | 0.36 | 1.8411 | 0.001 |  |  |  |  |  |
| Residual | 32 | 256922 | 0.63 |  |  |  |  |  |  |  |
| Total | 42 | 404742 |  |  |  |  |  |  |  |  |
